# Risk factors for acute kidney injury at presentation among children with CNS malaria: a case control study

**DOI:** 10.1186/s12936-022-04327-y

**Published:** 2022-11-01

**Authors:** Derby Tembo, Suzanna Mwanza, Chisambo Mwaba, Ifunanya Dallah, Somwe wa Somwe, Karl B. Seydel, Gretchen L. Birbeck

**Affiliations:** 1 Department of Paediatrics and Child Health, Chipata Central Hospital, Chipata, Zambia; 2 Dept. of Paediatric & Child Health, University of Zambia School of Medicine, Lusaka, Zambia; 3 Department of Neurology, University of Rochester, Rochester, NY 14642 USA; 4 University Teaching Hospitals, Neurology Research Office, Lusaka, Zambia; 5 Blantyre Malaria Project, Kamuzu University of Health Sciences, Blantyre, Malawi; 6 Dept. of Osteopathic Medical Specialties, College of Osteopathic Medicine, Michigan State University, East Lansing, MI USA; 7 University Teaching Hospitals Children’s Hospital, Lusaka, Zambia

**Keywords:** Coma, Seizure, Chronic kidney disease, Renal function, Sub-Saharan Africa

## Abstract

**Background:**

Recent research has established that acute kidney injury (AKI) is a common problem in severe paediatric malaria. Limited access to kidney diagnostic studies in the low resources settings where malaria is common has constrained research on this important problem.

**Methods:**

Enrolment data from an ongoing clinical trial of antipyretics in children with central nervous system (CNS) malaria, CNS malaria being malaria with seizures or coma, was used to identify risk factors for AKI at presentation. Children 2–11 years old with CNS malaria underwent screening and enrollment assessments which included demographic and anthropomorphic data, clinical details regarding the acute illness, and laboratory studies including creatinine (Cr), quantitative parasite count (qPC), quantitative histidine rich protein 2 (HRP2), lactate, and bilirubin levels. Children with a screening Cr > 106 µmol/l were excluded from the study due to the potential nephrotoxic effects of the study drug. To identify risk factors for AKI at the time of admission, children who were enrolled in the study were categorized as having AKI using estimates of their baseline (i.e. before this acute illness) kidney function and creatinine at enrollment applying the Kidney Disease: Improving Global Outcome (KDIGO) 2012 guidelines. Logistic regressions and a multivariate model were used to identify clinical and demographic risk factors for AKI at presentation among those children enrolled in the study.

**Results:**

465 children were screened, 377 were age-appropriate with CNS malaria, 22 (5.8%) were excluded due to Cr > 106 µmol/l, and 209 were enrolled. Among the 209, AKI using KDIGO criteria was observed in 134 (64.1%). One child required dialysis during recovery. Risk factors for AKI in both the logistic regression and multivariate models included: hyperpyrexia (OR 3.36; 95% CI 1.39–8.12) and age with older children being less likely to have AKI (OR 0.72; 95% CI 0.62–0.84).

**Conclusion:**

AKI is extremely common among children presenting with CNS malaria. Hyperpyrexia with associated dehydration may contribute to the AKI or may simply be a marker for a more inflammatory systemic response that is also affecting the kidney. Appropriate fluid management in children with CNS malaria and AKI may be challenging since generous hydration to support kidney recovery could worsen malaria-induced cerebral oedema in this critically ill population.

*Trial registration* https://clinicaltrials.gov/ct2/show/NCT03399318

## Background

Over the past decade, there has been increased appreciation that acute kidney injury (AKI) is a common complication of severe malaria in children [1, 2]. Available data estimate that about 24–59% of African children with severe malaria experience AKI [3]. The follow up of these survivors is challenging as very few of them will have kidney function assessed after discharge and the long-term effects of severe malaria on the kidney are just beginning to be explored. AKI is associated with increased mortality in critically ill patients. Recent research from Uganda has shown that 7.6% of children with severe malaria developed chronic kidney disease (CKD) at 1 year with AKI during the acute infection being a major risk factor for CKD [4]. AKI is associated with nephron damage and reduced functional kidney reserve over time and it is suggested that the risk of CKD may be influenced by the severity, number of episodes and the duration of AKI [3, 5].

The AKI guidelines from the Kidney Disease: Improving Global Outcome (KDIGO) consortium uses change in creatinine and urine output to define AKI [6]. Baseline (prior to acute illness) Cr is generally unknown in healthy children, especially in resource limited settings. Studies of paediatric kidney function in Africa are lacking even though the population is at high risk of AKI due to limited access to affordable and quality diagnostics. The diagnosis of malaria-associated AKI remains a challenge in sub-Saharan Africa due to lack of information on pre-malaria kidney function, limited access to laboratory facilities for Cr levels during the acute malaria infection and suboptimal resources and staffing for precise measurement of urine output. Researchers in Uganda have recently validated formulas to facilitate estimation of a child’s baseline normal creatinine value based upon age [7]. The purpose of this study was to identify the prevalence of and risk factors for AKI at presentation among children with central nervous system (CNS) malaria within the context of an ongoing neuroprotective clinical trial.

## Methods

A retrospective observational study was conducted using screening and admission data from an ongoing clinical trial of antipyretics in CNS malaria being conducted in Zambia at Chipata Central Hospital (CCH) and Malawi at Queen Elizabeth Central Hospital (QECH). (https://clinicaltrials.gov/ct2/show/NCT03399318).

Inclusion criteria for the clinical trial includes age 2–11 years old, having malaria based upon a positive rapid diagnostic test or *Plasmodium falciparum* on peripheral blood smear, and the presence of CNS manifestations of malaria including coma (Blantyre Coma Score (BCS) ≤ 2) or seizures. Because part of the clinical trial intervention includes ibuprofen, children were excluded if they presented with a Cr > 106 µmol/l. Other exclusion criteria were vomiting, circulatory collapse, jaundice or a total serum bilirubin > 265 µmol/l, having an allergy to paracetamol or nonsteroidal anti-inflammatory drugs, a contraindication to nasogastric tube insertion, or a history of liver disease, gastric ulcers, thrombocytopenia or haematological disorders.

Among eligible children, written informed consent from the parent/guardian was required. At the time of admission to the study site, enrolled children had extensive data collected including demographic characteristics, anthropomorphic data, clinical data regarding the acute infection including seizures prior to enrollment and administration of antipyretics, if applicable. Time of coma onset relative to presentation for care was captured only in Malawi. Laboratory data collected during the admission on enrolled children included quantitative parasite count (qPC), quantitative histidine rich protein 2 (HRP2), lactate, bilirubin, and packed cell volume (PCV). Admission data and blood samples were collected concurrent with treatment initiation and resuscitation, if needed. Fundoscopy to determine whether the malaria retinopathy was present was completed on admission at the Malawi site only [8, 9]. Discharge status was assessed by the clinician who evaluated the child on the day of discharge. A neurologic examination to identify neurologic sequelae, including visual impairments, severe hypotonia, or hemiparesis, was conducted the day of discharge. Outcomes included alive and well at discharge, alive with neurologic sequalae at discharge, or died.

Cr was determined based upon analysis of whole blood captured via finger prick using a STAT Sensor Creatinine Meter [10]. Quantitative parasite count (qPC) was determined by counting parasitized red cells on a thick smear stained with Field stain and comparing count to either PCV as an estimate of total red blood cell count or an estimated white blood cell count of 8,000 per/ul. Lactate and bilirubin were measured using point of care tests on whole blood (Lactate Pro, Arkay. Minneapolis, Minnesota and Reichert Technologies, Depew, New York) [11, 12]. Quantitative HRP2 was measured on stored plasma samples following the manufacturer’s protocol (Cellabs, Brookvale, Australia) with the modification of incubations being performed at 37 °C. The plates were analysed using an ELx800 reader at 450 nm (BioTek Instruments, Winooski, Vermont, USA). Plasma HRP-2 concentrations were calculated by comparing the results from patient samples with a standard curve generated from analysis of the recombinant stock. All results that fell outside the linear range were re-analysed after adjustment of dilution factors. Glucose was measured using whole blood obtained via finger prick and a handheld point-of care device [11]. HIV status was ascertained based upon any documented HIV antibody test result certified by a known authority or by antibody testing on the ward. For critically ill children, questions regarding developmental history and requests for HIV testing (if status not already known and documented in the child’s health passport), were often deferred until the child was stabilized.

Among admission characteristics, temperature was measured using a TempTraq® device placed in the axilla for measuring temperature every 2–4 min. Temperature was categorized as hypothermic < 36° Celsius (C), normothermic (36.0–37.0 °C), febrile (> 37, < 38.5 °C) or hyperpyrexic (≥ 38.5 °C). These categories were utilized rather than temperature as a continuous variable since hypothermia is uncommon but a risk factor for death [13] and hyperpyrexia is associated with more severe disease and poorer neurological outcomes [14, 15]. The association between high fevers and poorer neurological outcome may be due to the temperature-dependent nature of malaria parasite sequestration which may also have implications for malaria associated AKI [16]. Admission diagnosis was categorized as CNS malaria or cerebral malaria (CM) with CM representing the most severe manifestation of CNS malaria [17]. Any history of seizures before admission was categorized as none, single brief, or multiple or prolonged since duration of clinical seizure might be expected to contribute to dehydration and myoglobinuria—two potential contributors to AKI in malaria. When antipyretics were reported to have been given before admission, attempts were made to clarify the medication used. Anthropomorphic data were used to calculate weight for age, height for age and weight for length using World Health Organization Child Growth Standards [18].

Children enrolled in the clinical trial were categorized as having AKI using the KDIGO 2012 consortium guidelines which defines AKI based upon a change in Cr from baseline. For this analysis, the change in creatinine was determined by the difference between the admission creatinine and an estimated baseline creatinine, which was back-calculated using the Pottel age-based equation, where estimated glomerular filtration rate (GFR) = 107.3/(SCr/Q), (Q = 0.0270 * age + 0.2329) assuming a normal GFR of 120 mL/min per 1.73m2 as validated by recent studies in Ugandan children [7]. Using estimated baseline Cr, AKI at presentation was defined as a 1.5 fold ‘increase’ of creatinine on admission from estimated baseline. Although subsequent creatinine levels were obtained during the clinical trial, unblinding has not occurred and only data available at the time of admission before the study intervention was initiated were included in this report.

### Statistical analysis

For continuous variables, normally distributed variables were compared using an independent t-test and non-normal data were compared using the Mann–Whitney U tests. Chi-square, or Fisher’s exact tests were used when a cell count was less than 5, to identify differences in categorical variables. Bivariate logistic regressions were run to identify potential risk factors for AKI. Variables were included in a multivariate logistic regression if the information was captured at all study sites and the p-values from the bivariate logistic regression was less than or equal to 0.10. All statistical analyses were conducted using STATA Version 16.

## Results

**Fig. 1:**
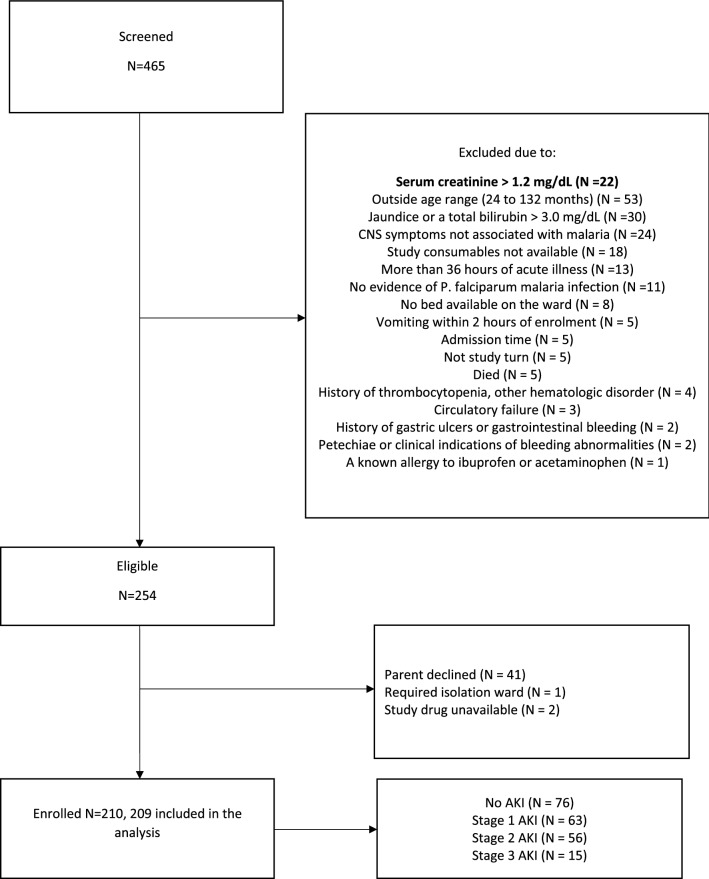
Enrollment flow

From January 7, 2019 to November 18, 2021, 465 children were screened, 377 were age-appropriate with CNS malaria or CM, 254 met eligibility criteria for clinical trial enrolment, and 210 were enrolled. Records for one child were lost during an infection control intervention on the research ward in Malawi so data from 209 children were available for analysis. See Fig. 1 for the screening and enrolment details.

Among age-appropriate children with CNS malaria screened for the clinical trial, 22 (5.8%) were excluded due to Cr > 106 µmol/l. All children excluded from the clinical trial for Cr > 106 µmol/l would have had AKI by the KDIGO criteria. See Table 1 for demographic and clinical details for the 209 children enrolled in the clinical trial and included in the AKI risk factor analyses. The mean age was 4.1 years old, 56.0% (n = 117) were male and 59.3% (n = 124) had CM (CNS malaria with coma). The prevalence of AKI among those enrolled using baseline estimates, the admission Cr and the KDIGO criteria was 134/209 (64.1%) with 63 (30.0%) having stage 1, 56 (26.8%), stage 2 and 15 (7.2%) stage 3, per KDIGO.

**Table 1:** Patient characteristics and risk factor analyses (n = 209)

	No AKI N = 75	Any AKI N = 134	All N = 209	Overall P-value	Bivariate OR (95% CI) p-value	Multivariate Model
*Demographics*						
Age, mean years (standard deviation (SD))	5.3 (2.3)	4.2 (1.8)	4.1 (2.1)	0.010	0.76 (0.66–0.88) p = 0.000	0.72 (0.62–0.84) p = 0.000
Gender, n (%) Male	40 (53.3)	77 (57.5)	117 (56.0)	0.56	1.18 (0.67–2.09) p = 0.56	
*History and clinical features at admission*						
Admission temperature, °C, n (%)				0.002		
Hypothermic, < 36.0	6 (8.0)	2 (1.5)	8 (3.8)		0.35 (0.06–2.02) p = 0.24	0.23 (0.04–1.39) p = 0.11
Normal, 36.0–37.0	17 (22.7)	16 (11.9)	33 (15.8)		–	**–**
Febrile, > 37.0, < 38.5	28 (37.3)	42 (31.3)	70 (33.4)		1.59 (0.69–3.67) p = 0.27	1.83 (0.75–4.50) p = 0.18
Hyperpyrexia, ≥ 38.5	24 (32.0)	74 (55.2)	98 (46.9)		3.28 (1.44–7.46) p = 0.005	3.36 (1.39–8.12) p = 0.007
Admission diagnosis, n (%)				0.31		
CNS malaria	34 (45.3)	51 (38.1)	85 (40.7)		–	
Cerebral malaria	41 (54.7)	83 (61.9)	124 (59.3)		1.35 (0.76–2.39) p = 0.31	
Developmental delay before illness, n (%)	4 (5.3)	7 (5.2)	11 (5.3)	0.97	0.97 (0.28–3.46) p = 0.97	
Coma duration prior to admission, hrs^1^, mean (SD)	N = 24 24.2 (18.5)	N = 73 22.1 (17.3)	N = 97 22.6 (17.6)	0.61	0.99 (0.97–1.02) p = 0.61	
History of seizures with this illness prior to enrolment, n (%)				0.21		
None	8 (10.7)	27 (20.2)	35 (16.8)		–	
Single and brief	9 (12.0)	15 (11.2)	24 (11.5)		0.49 (0.16–1.55) p = 0.23	0.35 (0.10–1.24) p = 0.10
Multiple or prolonged	58 (77.3)	92 (68.7)	150 (71.8)		0.47 (0.20–1.10*)* p = 0.083	0.43 (0.17–1.12) p = 0.084
Clinical retinopathy^1^, n (%)	N = 24 10 (41.6)	N = 73 43 (58.9)	N = 97 53 (54.6)	0.14	1.97 (0.95–4.09) p = 0.07	
Antipyretics prior to enrolment, n (%)	66 (88.0)	118 (88.1)	184 (88.0)	0.99	1.01 (0.42–2.40) p = 0.99	
Paracetamol as antipyretic, n (%)	N = 66 62 (93.9)	N = 118 112 (94.9)	N = 184 174 (94.5)	0.78	1.20 (0.33–4.43) p = 0.78	
Anticonvulsants prior to enrolment, n (%)	37 (49.3)	74 (55.2)	111 (53.1)	0.41	1.27 (0.72–2.23) p = 0.41	
Outcome, n (%)	N = 75	N = 134	N = 209	0.12		
Alive and well	70 (93.3)	112 (83.6)	182 (87.1)			
Alive with neurologic sequelae	2 (2.7)	13 (9.7)	15 (7.2)			
Died	3 (4.0)	9 (6.7)	12 (5.7)			
Weight for Age, n (%)	N = 71	N = 133	N = 204	0.44		
Normal	51 (71.8)	99 (74.4)	150 (73.5)		–	
Underweight	17 (23.9)	24 (18.1)	41 (20.1)		0.73 (0.36–1.47) p = 0.38	
Severely underweight	3 (4.2)	10 (7.5)	13 (6.4)		1.72 (0.45–6.52) p = 0.43	
Height for Age, n (%)	N = 54	N = 118	N = 172	0.65		
Normal	44 (81.5)	90 (76.3)	134 (77.9)		–	
Stunted	7 (13.0)	22 (18.6)	29 (16.9)		1.54 (0.61–3.87) p = 0.36	
Severely stunted	3 (5.6)	6 (5.1)	9 (5.2)		0.98 (0.23–4.09) p = 0.98	
Weight for length, n (%)	N = 26	N = 90	N = 116	0.13		
Normal	25 (96.2)	72 (80.0)	97 (83.6)		–	
Wasting	1 (3.9)	11 (12.2)	12 (10.3)		2.70 (0.46–15.68) p = 0.27	
Severe wasting	0 (0.0)	7 (7.8)	7 (6.0)		5.26 (0.29–95.70) p = 0.26	
*Laboratory findings at admission*						
qPC	N = 74 620	N = 125 440	N = 199 480 Range 0–2,806,000	p = 0.49	0.97 (0.89–1.06) p = 0.49	
HRP2	N = 54 97	N = 88 183	N = 199 165 Range 0.2^2^–3305	p = 0.30	1.11 (0.91–1.36) p = 0.29	
Bilirubin on admission, mg/dL, mean (SD)	0.77 (0.74)	0.77 (0.70)	0.77 (0.72)	0.96	0.99 (0.67–1.47) p = 0.96	
Lactate on admission, mmol/l, mean (SD)	5.1 (4.1)	4.7 (4.9)	4.8 (4.8)	0.45	0.98 (0.92–1.03) p = 0.48	
PCV on admission, mean (SD)	28.4 (6.0)	N = 133 27.7 (7.4)	N = 208 27.97 (6.9)	0.48	0.99 (0.94–1.03) p = 0.48	
HIV status, n (%)	N = 71	N = 132	N = 203	0.66		
Positive	3 (4.2)	4 (3.0)	7 (3.5)		0.71 (0.15–3.26) p = 0.66	
Admission glucose, n (%)				0.50		
Hypoglycaemia	4 (5.3)	4 (3.0)	8 (3.8)		0.51 (0.12–2.11) p = 0.35	
Normal	48 (64.0)	95 (70.9)	143 (68.4)		–	
Hyperglycaemia	23 (30.7)	35 (26.1)	58 (27.8)		0.77 (0.41–1.44) p = 0.41	

Available only in participants at QECH

Since malaria rapid diagnostic test positive, HRP2 = 0 imputed to be lowest detectable limit of 0.2

Table 1 also details AKI risk factors analyses. Risk factors for AKI identified in the bivariate analyses included age (OR 0.76; 95% CI 0.66–0.88, p = 0.000) and being hyperpyrexic on admission (OR 3.28; 95% CI 1.44–7.46, p = 0.005). In a multivariate model including age, presence of hyperpyrexia, and seizures prior to admission, age (OR 0.72; 95% CI 0.62–0.84; p-0.000) and hyperpyrexia (OR 3.36; 95% CI 1.39–8.12, p = 0.007) remained risk factors.

## Discussion

Among 377 children aged 2–11 years with CNS malaria or CM who were screened for enrollment in the clinical trial, 156 (41.4%) had evidence of AKI at presentation based upon screening Cr > 106 µmol/l OR applying the KDIGO criteria for AKI using estimates of baseline kidney function compared to admission kidney function among those enrolled in the clinical trial. This is similar to other recent reports on AKI in African children with severe malaria [3]. Reasons for the growing body of literature indicating that malaria-related kidney injury is a major problem in malarial regions could be due to uptake of the standardization of the definition of AKI by the KDIGO 2012 consortium [19] and/or the changing epidemiology of malaria. As malaria control programmes are implemented, many areas have changed from hyper-transmission zones to endemic regions meaning that a lower proportion of the population will be immune and so are at higher risk of severe forms of malaria. Such an association between transmission intensity and clinical manifestations of severe falciparum have been previously noted for cerebral malaria [15].

The primary limitation of this study is that the inclusion criteria for the clinical trial and thus the analytic sample for AKI risk factor analyses excluded children with Cr > 106 µmol/l, elevated bilirubin and/or vomiting. As such, the population included in the risk factor analyses is not fully representative of all children with CNS malaria. Nevertheless, the risk factor analysis provides some insights. Potential pathophysiologic mechanisms for AKI in malaria include haemoglobinuria, parasite sequestration, volume depletion, endothelial activation, microcirculatory dysfunction, inflammation and immune activation [3, 20, 21]. In the present study, haemoglobin and bilirubin levels, possible proxies for the quantification of the severity of haemolysis [22], were not identified as risk factors of AKI, but children with visible jaundice or bilirubin > 265 µmol/l were actively excluded from clinical trial enrolment. Malaria parasite biomass, as assessed by quantifying HRP-2 at admission, was also not associated with AKI in contrast to some recent studies which have showed an association [21, 23]. In this study, being older was found to be a protective factor (OR 0.76; 95% CI 0.66–0.88, p = 0.01). It is known that in endemic regions, malaria is more likely to affect children < 5 years who have not yet developed established specific acquired immunity [24, 25]. Whether partial immunity might impact kidney vulnerabilities to malaria among children is unknown. Recruitment criteria excluded children with vomiting and/or circulatory collapse, thus dehydration as a risk factor could not be reasonably evaluated. A Ugandan study found that vomiting was associated to AKI [23]. Retinopathy in CM represents direct evidence of microcirculatory obstruction which is linked to red cell sequestration [26] and some autopsy studies have shown evidence of red cell sequestration in kidney microvasculature [27]. In this study, retinopathy was not a significant risk for AKI. (OR 1.97; 95% CI 0.95–4.05, p = 0.07). Having hyperpyrexia with a temperature of  > / = 38.5 °C on admission was an AKI risk factors. Fever is a clinical manifestation of malaria attributed to the activation of the inflammatory system [28]. Several cytokines have been associated with disease syndromes such as CM [29–31], but studies specifically showing a correlation between fever or pyrogen levels and the risk of malaria-associated kidney injury are scanty [32].

## Conclusion

In resource limited malaria endemic areas, children with CNS malaria at highest risk of AKI need to be identified for opportune supportive care as kidney replacement therapy is not easily accessible [33, 34]. Unfortunately, supportive care with generous hydration may worsen neurologic morbidity since it might increase cerebral oedema and thus mortality [35]. Raising awareness of AKI and its risk factors in complicated pediatric malaria among primary health care providers to facilitate referral to higher centres of care is feasible and warranted [33]. Studies are also needed to determine optimal fluid management of comorbid AKI and CNS malaria.

## Data Availability

The datasets used and/or analysed during the current study are available from the corresponding author on reasonable request.
